# A Novel Racing Array Transducer for Noninvasive Ultrasonic Retinal Stimulation: A Simulation Study

**DOI:** 10.3390/s19081825

**Published:** 2019-04-17

**Authors:** Yanyan Yu, Zhiqiang Zhang, Feiyan Cai, Min Su, Qiuju Jiang, Qifa Zhou, Mark S. Humayun, Weibao Qiu, Hairong Zheng

**Affiliations:** 1 Paul C. Lauterbur Research Center for Biomedical Imaging, Shenzhen Institutes of Advanced Technology, Chinese Academy of Sciences, Shenzhen 518055, China; yy.yu@siat.ac.cn (Y.Y.); zq.zhang@siat.ac.cn (Z.Z.); fy.cai@siat.ac.cn (F.C.); min.su@siat.ac.cn (M.S.); qj.jiang@siat.ac.cn (Q.J.); 2 Shenzhen Key Laboratory of Ultrasound Imaging and Therapy, Shenzhen 518055, China; 3 Ginsburg Institute for Biomedical Therapeutics, Roski Eye Institute and Biomedical Engineering, University of Southern California, Los Angeles, CA 90033, USA; qifazhou@usc.edu (Q.Z.); humayun@med.usc.edu (M.S.H.)

**Keywords:** racing array transducer, ultrasonic retinal stimulation, noninvasive neurostimulation, patterned stimulation

## Abstract

Neurostimulation has proved to be an effective method for the restoration of visual perception lost due to retinal diseases. However, the clinically available retinal neurostimulation method is based on invasive electrodes, making it a high-cost and high-risk procedure. Recently, ultrasound has been demonstrated to be an effective way to achieve noninvasive neurostimulation. In this work, a novel racing array transducer with a contact lens shape is proposed for ultrasonic retinal stimulation. The transducer is flexible and placed outside the eyeball, similar to the application of a contact lens. Ultrasound emitted from the transducer can reach the retina without passing through the lens, thus greatly minimizing the acoustic absorption in the lens. The discretized Rayleigh–Sommerfeld method was employed for the acoustic field simulation, and patterned stimulation was achieved. A 5 MHz racing array transducer with different element numbers was simulated to optimize the array configuration. The results show that a 512-element racing array is the most appropriate configuration considering the necessary tradeoff between the element number and the stimulation resolution. The stimulation resolution at a focus of 24 mm is about 0.6 mm. The obtained results indicate that the proposed racing array design of the ultrasound transducer can improve the feasibility of an ultrasound retinal prosthesis.

## 1. Introduction

Retinitis pigmentosa and age-related macular degeneration can cause vision loss in people [1]. An implantable retinal prosthesis based on electrical retinal stimulation has been developed and applied for the restoration of vision in patients [2,3]. Although the patient has lost part of their sight, some neural cells in the retina remain alive and can be stimulated directly by electrodes attached to the retina [3,4]. A retinal prosthesis provides a feasible solution for visual restoration. The highly invasive procedure performed during the implantation surgery, however, makes the device high-cost and high-risk [5]. Therefore, a noninvasive method for visual restoration is of great interest and strongly needed in society.

There are several noninvasive neurostimulation methods, including direct current stimulation and magnetic stimulation [6]. However, the spatial resolution of these two methods is poor (about one centimeter) and is thus not suitable for retinal stimulation. As a mechanical wave, ultrasound has recently drawn great attention for its potential use in noninvasive neurostimulation because it can pass through human tissue, even high-density tissue, such as the human skull [7]. Several noninvasive neurostimulation studies have applied ultrasound using different models, including neural cells [8], small animals [9,10,11], and primates [12], as well as human subjects [13]. Ultrasound for retinal stimulation has also been proposed by several research groups. Two-dimensional-patterned stimulation can be achieved by multi-focal ultrasonic distributions with an array transducer [14], and very-high-frequency ultrasound was proposed for high-resolution retinal stimulation [15]. Our group has demonstrated that ultrasound can activate rat retinal ganglion cells in vitro successfully [16]. However, previous works on neurostimulation have been based on large-sized single-element transducers or array transducers, which are not feasible for clinical retinal stimulation. In addition, a single-element transducer can generate only one focus at a time, and the focus position is hard to change, so its use for practical applications is limited. Multiple-focus stimulation can be achieved using an array transducer, and the stimulation can be targeted to different areas of living tissue simultaneously [17]. Therefore, a small array transducer with a contact lens shape was proposed previously as a wearable retina prosthesis [18], providing an easy way to implement an ultrasound retinal prosthesis. However, lens tissue in the eyeball has high acoustic absorption (7.8 dB/cm @10 MHz) [19], so most of the ultrasound energy is likely to be absorbed by the lens tissue, especially when high-frequency ultrasound is used. The absorption causes an elevation in temperature, which can be harmful to the eye.

To overcome this problem, a new racing array transducer for a noninvasive ultrasonic retinal prosthesis is proposed in this work. The shape of the transducer is concave, similar to that of a contact lens, and the center of the transducer is hollow. All the array elements are aligned along the concave surface. The transducer is flexible and can contact the eyeball directly, and the ultrasonic waves can reach the retinal tissue without passing through the lens tissue, which minimizes the damage to the lens. Here, we report a simulation study that was conducted to evaluate the performance of the racing array transducer for noninvasive ultrasound stimulation.

## 2. Method

### 2.1. Design of Array Transducer

Our previous study [18] proposed an array transducer for a retinal prosthesis. It mimics a contact lens that covers the whole pupil with 256 ultrasonic elements. Although patterned neurostimulation can be achieved, the lens tissue is likely to absorb energy from the acoustic waves, which are typically harmful to living tissue.

**Figure 1:**
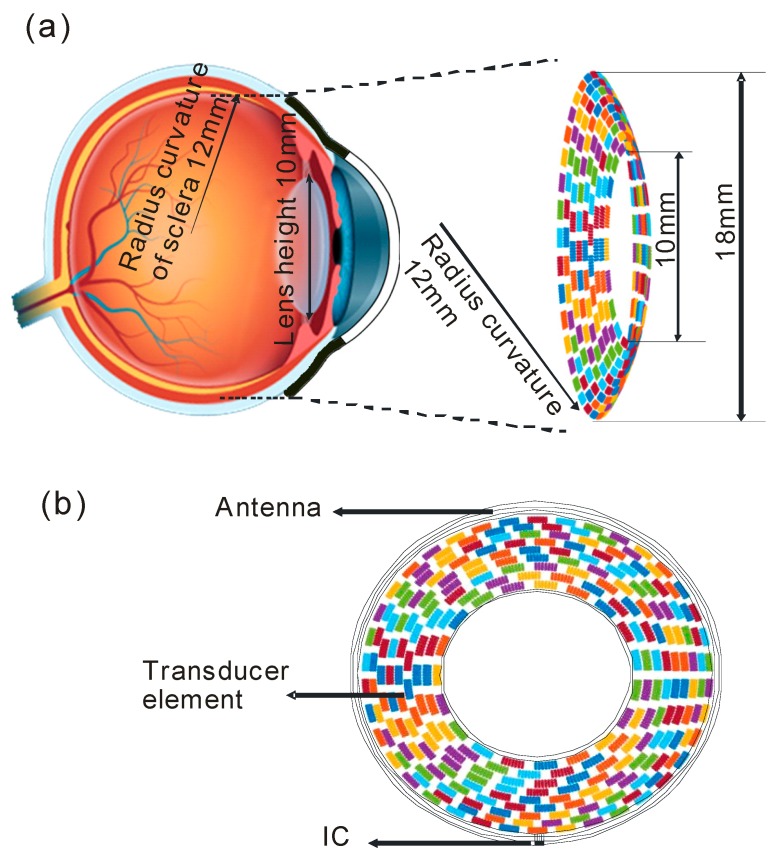
The proposed racing array device in this study for ultrasonic stimulation. (**a**) Lateral view of the device; (**b**) Top view of the device.

Therefore, a novel structure is proposed that uses a circular racing array for the stimulation, as shown in Figure 1. As illustrated in Figure 1a, which shows the anatomical structure of the human eye, the outer diameter and the inner diameter of the racing array transducer were designed to be 10 mm and 18 mm, respectively. The focal length of the ultrasound stimulation was set to be about 24 mm, which is the typical diameter of an adult eyeball. (The human adult eye is about 24.2 mm in the transversal direction, 23.7 mm in the sagittal direction, and 22.0–24.8 mm in the axial direction [20]). The radius curvature of the racing array was set to be 12 mm, which roughly equals the radius curvature of the human eyeball. To implement a fully functional device, an antenna and an integrated circuit (IC) can be arranged in the device, as shown in Figure 1b, to receive and process signals from a camera placed outside the device [18]. The proposed design offers a new method in which the ultrasonic wave can reach the retina without passing through the lens. In this case, higher-frequency ultrasound can be used for retinal stimulation. As a result, the stimulation resolution increases, and more complex patterns can be presented.

Six different types of transducers were investigated in this work to find a suitable solution for the racing array transducer. The physical parameters of the six different types of transducers are listed in detail in Table 1. Two ultrasound frequencies were simulated (2.5 MHz and 5 MHz), and the number of elements ranged from 256 to 1024. The element sizes are listed in the third column of Table 1.

### 2.2. Simulation Setup

**Figure 2:**
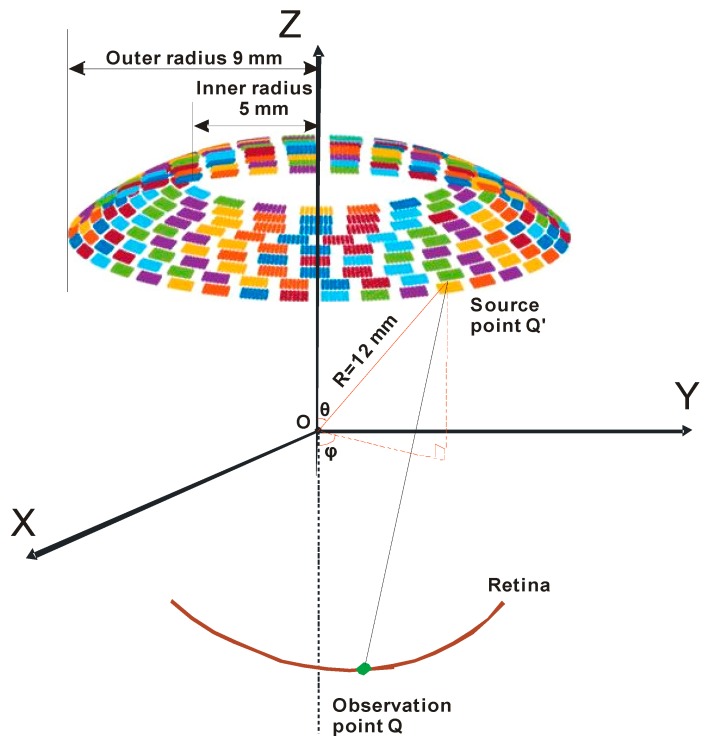
Configuration and geometry of the racing array transducer for the calculation of the acoustic field.

To create patterned stimulation in the retina, a method based on discretized Rayleigh–Sommerfeld [21] was applied to generate a matrix form propagation operator in this study. The configuration and geometry for calculating the acoustic field are shown in Figure 2. The pressure of an acoustic waveform from a source point Q′ on the racing array to the observation point Q can be described as

(1) $$p\left(Q\right)=\frac{j\rho ck}{2\pi }\int_{S}u\left(Q\prime \right)\frac{e^{j\left(\omega t-k\left|Q-Q^{\prime }\right|\right)}}{\left|Q-Q^{\prime }\right|}dS$$

where p(Q) is the acoustic pressure at the observation point, ρ is the density of the medium, ω and λ are the frequency and wavelength of the ultrasound, respectively, S indicates the surface of the source area, and u is the excitation of the source point on the transducer. If we suppose that the number of elements on the transducer is N, then the acoustic pressure of the observation point Q can be rewritten as

(2) $$p\left(Q\right)=\frac{j\rho ck}{2\pi }\sum_{n=1}^{N}u_{n}\int_{S_{n}}\frac{e^{j\left(\omega t-k\left|Q-{Q^{\prime }}_{n}\right|\right)}}{\left|Q-{Q^{\prime }}_{n}\right|}dS_{n}$$

where $u_{n}$ is the excitation of the nth element, and ${Q^{\prime }}_{n}$ indicates the source points on the nth element. Then,

(3) $$dS_{n}=R^{2}\sin\phi d\phi d\theta$$

where R is the radial distance, θ is the azimuthal angle, and ϕ is the polar angle. Their position is shown on the spherical coordinate system in Figure 2. If we suppose that the pressure at the mth observation point is $p\left(Q_{m}\right)$, then it can be expressed as

(4) $$p\left(Q_{m}\right)=\frac{j\rho ck}{2\pi }\sum_{n=1}^{N}u_{n}\int_{S_{n}}\frac{e^{j\left(\omega t-k\left|Q_{m}-{Q^{\prime }}_{n}\right|\right)}}{\left|Q_{m}-{Q^{\prime }}_{n}\right|}dS_{n}$$

where $p=\left[p\left(Q_{1}\right),p\left(Q_{2}\right),\ldots ,\;p\left(Q_{M}\right)\right]$ is a vector that represents the acoustic pressure at observation points in the retina, and $U=\left[u_{1},u_{2},\ldots ,u_{N}\right]$ indicates a vector that includes the excitation of *N* elements on the transducer.

Here, the term $\frac{j\rho ck}{2\pi }\int_{S_{n}}\frac{e^{j\left(\omega t-k\left|Q_{m}-{Q^{\prime }}_{n}\right|\right)}}{\left|Q_{m}-{Q^{\prime }}_{n}\right|}$ is defined as the forward propagation operator H and is expressed as

(5) $$H=\;\frac{j\rho ck}{2\pi }\int_{S_{n}}\frac{e^{j\left(\omega t-k\left|Q_{m}-{Q^{\prime }}_{n}\right|\right)}}{\left|Q_{m}-{Q^{\prime }}_{n}\right|}.$$

Therefore, Equation (4) can be rewritten as

(6) p=HU.

The solution of the excitation term U can be obtained by the minimum norm least-square method using the following equation [21]:

(7) $$\hat{U}={\left(H^{*}\right)}^{t}{\left(H{\left(H^{*}\right)}^{t}\right)}^{-1}p$$

where $H^{*}$ is the pseudoinverse of the term H and can be derived by the spectral value decomposition (SVD). ${\left(H^{*}\right)}^{t}$ is the conjugate transpose of $H^{*}$ and indicates the backward propagation operator from the observation point. 

The excitation efficiency was improved by an iterative weighting method:

(8) $$\hat{U}=W{\left(H^{*}\right)}^{t}{\left(HW{\left(H^{*}\right)}^{t}\right)}^{-1}p$$

where W is a positive weighting matrix with *N* ×N elements. The matrix was set as an identity matrix, and then W was obtained by an iterative weighting algorithm [22].

### 2.3. Acoustic Field Computation

The ultrasound-focused pattern on the retina can be derived by taking the integral of the acoustic pressure from all of the source points. Figure 2 presents the diagram for calculating the ultrasound field. Each array element is divided into infinitesimal source points. The area of the infinitesimal element is described by Equation (3). Therefore, the acoustic pattern on the retina can be described as

(9) $$p\left(x,y,z,t\right)=\frac{j\rho ck}{2\pi }\;\sum_{n=1}^{N}\hat{U_{n}}e^{j\omega t}\iint \frac{e^{-jkL}}{L}R^{2}sin\phi d\phi d\theta$$

where *L* represents the distance from the infinitesimal source to the observation point in the acoustic pattern. The term p(x,y,z,t) is the pressure at the observation point at time *t*. Therefore, the spatial-peak pulse-average intensity *I*(*x*, *y*, *z*) can be expressed as [23]

(10) $$I\left(x,y,z\right)=\frac{p\times p^{*}}{2\rho c}$$

where p and $p^{*}$ are the acoustic pressure and its conjugate, respectively.

## 3. Results

### 3.1. Resolution by Different Ultrasonic Frequencies

**Figure 3:**
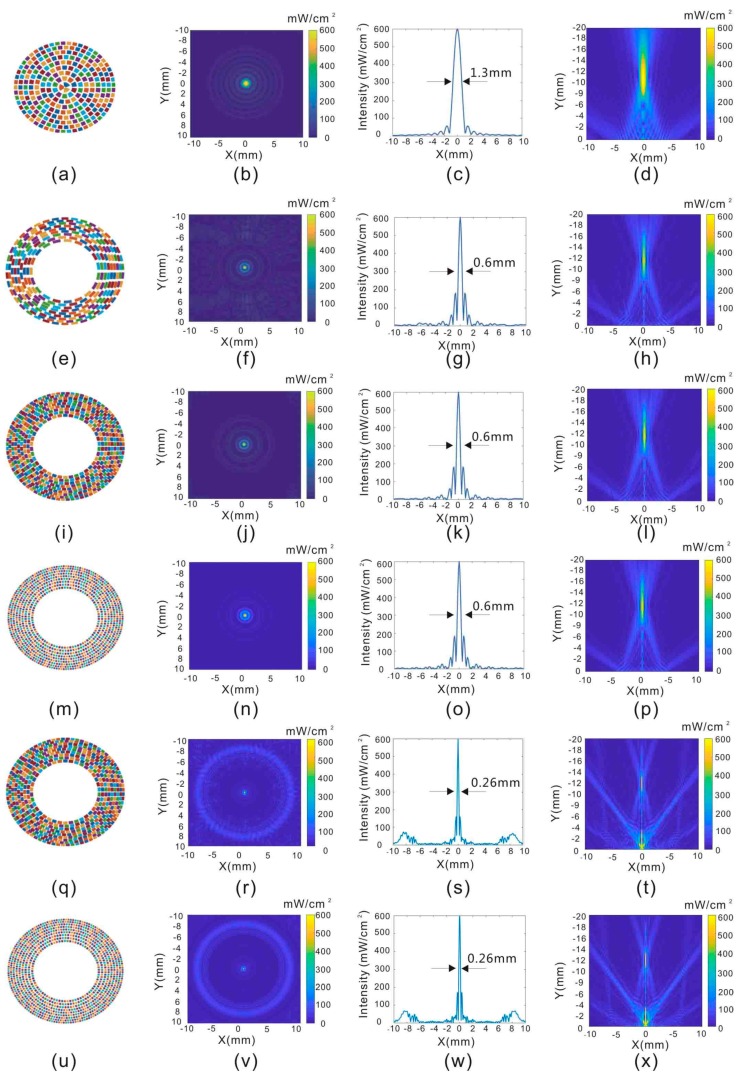
The first column shows six different types of transducers. The transducers have the following parameters: (**a**) 2.5 MHz, 256 elements; (**e**) 5 MHz, 256 elements; (**i**) 5 MHz, 512 elements; (**m**) 5MHz, 1024 elements; (**q**) 10 MHz, 512 elements; (**u**) 10 MHz, 1024 elements. (**b**,**f**,**j**,**n**,**r**,**v**) in the second column show the two-dimensional (2D) acoustic intensity field in the X–Y plane with corresponding elements in the first column. (**c**,**g**,**k**,**o**,**s**,**w**) in the third column show the resolution of the focal point generated by the elements in the first column. (**d**,**h**,**l**,**p**,**t**,**x**) in the fourth column shows the 2D acoustic intensity field in the X–Z plane created by the elements in the first column. The units of acoustic intensity are based on spatial-peak temporal averaging intensity.

The parameters of the six types of transducers listed in Table 1 were evaluated in this study. Throughout this study, the adult eyeball was regarded as a sphere with a diameter of 24 mm. The center of the eyeball is set at the origin of the coordinates in Figure 2. The resolutions at all of the coordinates were evaluated by setting a single focal point located at (0, 0, −12), which is where the posterior of the retina is located in the coordinate system. The resolution can be quantified by estimating the full-width at half maximum (FWHM) of the profile. The Type A transducer has the same structure (256 elements, 2.5 MHz) as that in our previous published work [18]. The resolution of a single focal point is about 1.3 mm, which is shown in Figure 3c. The Type B transducer is one of the proposed racing array transducers, and it has 256 elements in a circular arrangement. Although the working frequency of the Type B transducer was increased to 5 MHz, it has limited influence on the lens tissue since the path of ultrasound is not through the lens. It can be observed in Figure 3g that the resolution of the 5 MHz transducer with 256 elements is improved to 0.6 mm. However, the background noise is also increased, as shown in Figure 3f. As the number of elements in the transducer increases to 512, the noise disappears, and the resolution remains 0.6 mm, as shown in Figure 3k. Considering these simulation results, 512 array elements and a 5 MHz ultrasound frequency were considered to be a good combination for the racing array transducer. Actually, our group previously proved that 5 MHz ultrasound provides effective neurostimulation [24]. Therefore, 5 MHz ultrasound was selected for further studies.

### 3.2. Construction of Multiple Focal Points

**Figure 4:**
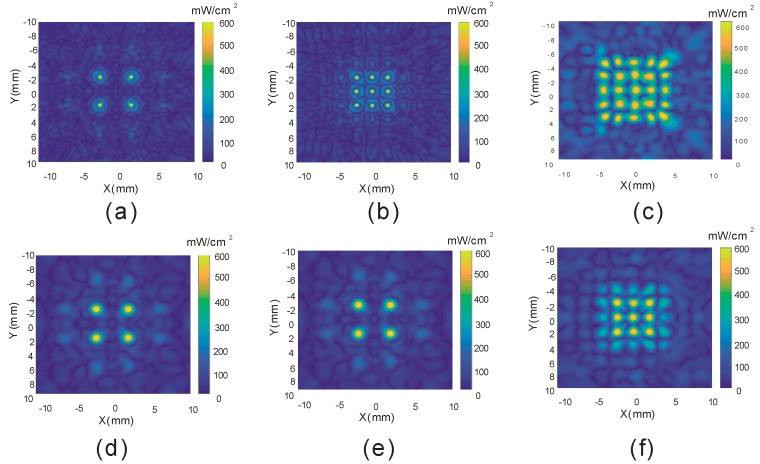
Acoustic intensity distribution of multiple points in the X–Y plane: (**a**–**c**) are the intensity fields derived from a racing transducer of 5 MHz with 512 elements; (**d**–**f**) are the intensity fields derived from a racing transducer of 2.5 MHz with 256 elements.

The method based on discretized Rayleigh–Sommerfeld [20] allows us to create complex ultrasound patterns at different positions of the retina instantaneously. To verify this function, the electrical excitation term $\hat{U}$ for each transducer element in Equation (7) was derived by setting the pressure value $p=\left[p\left(Q_{1}\right),p\left(Q_{2}\right),\ldots ,\;p\left(Q_{M}\right)\right]$ at the multiple observation points in Equation (4). Figure 4a–c are the intensity fields derived from the 5 MHz racing array transducer with 512 elements and 4, 9, and 25 focal points, respectively. For comparison, the intensity field derived from a traditional focused transducer (2.5 MHz with 256 elements) [18] is shown in the second row of Figure 4. The average FWHM resolutions at the focal points generated by the 5 MHz and 2.5 MHz racing arrays are 0.6 mm and 1.3 mm, respectively. It can be observed that the racing array with a 5 MHz frequency and 512 ultrasonic elements has a better performance in terms of resolution and complex pattern generation.

**Figure 5:**
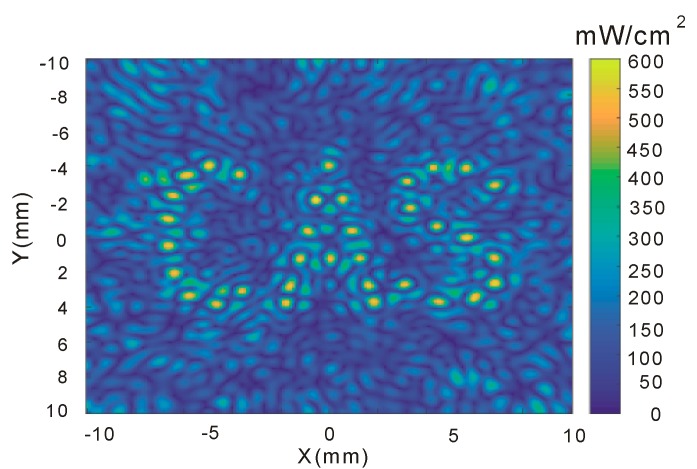
Acoustic intensity distribution of the “CAS” (Chinese Academy of Sciences) pattern in the X–Y plane.

A more complex pattern with three characters, “CAS”, which is an abbreviation of Chinese Academy of Sciences, can also be constructed by the proposed racing array transducer. The derived ultrasound field “CAS” in the X–Y plane is shown in Figure 5.

### 3.3. Patterns at Different Depths

**Figure 6:**
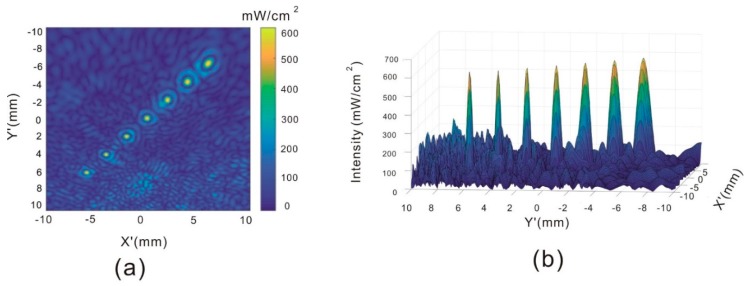
Acoustic intensity distribution at different depths. (**a**) Acoustic intensity of seven focal points in the across section; (**b**) 3D acoustic intensity of seven focal points in the across section.

Retinal tissue is not fully flat, and this can cause some variation in the resolution. Therefore, we evaluated the performance of the racing array transducer in the creation of patterns at different depths. The seven points from the bottom left to the top right corner in Figure 6a are located at (−6, 6, −6), (−4, 4, −8), (−2, 2, −10), (0, 0, −12), (2, −2, −14), (4, −4, −16), (6, −6, −18) in the axial direction in the coordinates of Figure 2. The coordinate set (0, 0, 0) is the center of the eyeball, shown as O in Figure 2. The posterior of the eyeball is at (0, 0, −12). The intensity cross-section through the seven points is shown in Figure 6a. The FWHM resolutions of the seven points are 0.82 mm, 0.72 mm, 0.62 mm, 0.60 mm, 0.44 mm, 0.44 mm, and 0.44 mm, respectively. It can be observed that the resolution increases as the distance increases between the focal point and the X–Y plane, but all seven focal points can be clearly captured by the cross-section.

## 4. Discussion

A novel racing array transducer is proposed for noninvasive retina stimulation. The proposed transducer is flexible and placed outside the eyeball, similar to the application of a contact lens. The lens tissue is highly absorptive of acoustic waves; therefore, it is not safe to pass ultrasonic waves through the lens tissue. The proposed new design employs a circular array, and the lens tissue can be avoided during retinal stimulation. The transducer has a contact lens shape, and the flexible nature of the device results in its easy application for noninvasive retina stimulation.

The frequency of ultrasound can be increased to a higher frequency since there is no lens tissue in the acoustic wave path, and the stimulation resolution can be improved. However, one issue we have to compromise on is the grating lobe. A grating lobe will occur during the beam steering when the pitch size of an array is larger than the wavelength, and it will generate an artifact during the neurostimulation process. Therefore, reducing the grating lobe is very important for the design of a racing array transducer. Since the wavelength is inversely proportional to the ultrasound frequency, higher-frequency ultrasound requires a smaller element size. The racing array is the size of a normal contact lens, and if 10 MHz ultrasound is selected, the total number of elements in the array will be more than 3000 if we keep the pitch to a wavelength level. This is a huge number, which makes the transducer difficult to fabricate. Lower-frequency ultrasound is more feasible for practical applications. Even a lower frequency can stimulate unwanted areas, especially in depth. These unwanted effects can be overcome by adjusting the power intensity of the ultrasound since there is a power threshold for neuromodulation (about 0.25 W/cm^2^, [15]). The power of the ultrasound should be controlled to be just a little bit higher than that threshold to minimize the depth artifact of ultrasonic neuromodulation.

**Figure 7:**
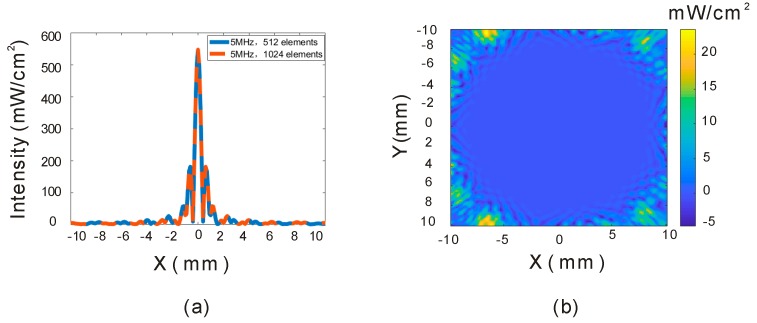
Acoustic intensity comparison between 512- and 1024-element transducers. (**a**) Acoustic intensity through the focal points. (**b**) Intensity difference (X–Z plane) between two array transducers.

There is not much difference between using 512 and 1024 elements for the 5 MHz array. Figure 7a shows the acoustic intensities through the focal points, and the data are almost the same, especially in the focal zone. Figure 7b shows the subtraction of Figure 3j,n, and it can be seen that the difference is mainly distributed in the peripheral area. However, the fabrication process is more difficult for 1024 elements, so 512 is a better choice for this application.

According to the Food and Drug Administration (FDA) regulation [25], the acoustic intensity on the eye should be lower than 50 mW/cm^2^, which is much smaller than the threshold for other organs, which can tolerate 720 mW/cm^2^. The main reason for the restriction is the absorption by the lens tissue. However, the proposed racing array transducer can stimulate the retinal tissue without passing through the lens tissue. Therefore, the intensity level can be higher than the normally required limit. The data shown in this manuscript are from simulations rather than experiments. Fabricating such a racing array transducer takes substantial engineering work, so it is necessary to simulate all the parameters first to determine the optimized configuration of the actual transducer. The simulated results show that the racing array transducer has a better performance than the traditional transducer.

## 5. Conclusions

A novel racing array transducer with a contact lens shape is proposed for a retinal prosthesis. In this approach, higher-frequency ultrasound can be used because the ultrasound emitted from the racing array transducer can reach the retina without passing through the lens. The simulation results show that the proposed design can improve the resolution from 1.3 mm to 0.6 mm compared with the design from previous work. A more complex acoustic pattern can also be achieved. The results of this study offer theoretical data for the next step, which is the fabrication process of the noninvasive ultrasound retinal prosthesis.

## Figures and Tables

**Table 1:** The physical parameters of array transducers simulated in this study.

	Frequency (MHz)	Element Number	Element Size (mm × mm)	Inner Diameter (mm)	Outer Diameter (mm)
Type (a)	2.5	256	0.61 × 0.58	0	14
Type (e)	5	256	0.28 × 0.94	10	18
Type (i)	5	512	0.37 × 0.50	10	18
Type (m)	5	1024	0.22 × 0.30	10	18
Type (q)	10	512	0.37 × 0.50	10	18
Type (u)	10	1024	0.22 × 0.30	10	18
